# Supplementary material for: Mapping the evolution of fertility support policies in China: A content and instrumental analysis
Source: PLoS One. 2025 Oct 9;20(10):e0332137. doi: 10.1371/journal.pone.0332137 (PMC12510515; doi:10.1371/journal.pone.0332137)
Supplement: S1 Appendix — (ZIP) [file pone.0332137.s001.zip › S1 Appendix. 226 original policy documents/65-国务院办公厅转发人口计生委扶贫办关于进一步做好人口计生与扶贫开发相结合工作若干意见的通知(FBM-CLI-2-167107).doc]

国务院办公厅转发人口计生委扶贫办关于进一步做好人口计生与扶贫开发相结合工作若干意见的通知

**发布部门：** [国务院办公厅](https://www.pkulaw.com/law/chl?Aggs.IssueDepartment=601) [机构沿革](javascript:void(0))

**发文字号：**国办发〔2012〕10号

**发布日期：**2012.02.05

**实施日期：**2012.02.05

**时效性：** [现行有效](https://www.pkulaw.com/law/chl?Aggs.TimelinessDic=01)

**效力级别：** [国务院规范性文件](https://www.pkulaw.com/law/chl?Aggs.EffectivenessDic=XC0203)

**法规类别：** [计划生育管理](https://www.pkulaw.com/law/chl?Aggs.Category=09705) [扶贫救灾救济](https://www.pkulaw.com/law/chl?Aggs.Category=01504)

国务院办公厅转发人口计生委扶贫办关于进一步做好人口计生与扶贫开发相结合工作若干意见的通知
（国办发〔2012〕10号）

各省、自治区、直辖市人民政府，国务院各部委、各直属机构：
　　人口计生委、扶贫办《关于进一步做好人口计生与扶贫开发相结合工作的若干意见》已经国务院同意，现转发给你们，请认真贯彻执行。

国务院办公厅
二○一二年二月五日

关于进一步做好人口计生与扶贫开发相结合工作的若干意见
（人口计生委、扶贫办）

　　为深入贯彻《[中华人民共和国国民经济和社会发展第十二个五年规划纲要](https://www.pkulaw.com/chl/603c7a7f2158b52bbdfb.html)》和《中国农村扶贫开发纲要（2011-2020年）》精神，进一步做好新形势下人口计生与扶贫开发相结合工作，提出如下意见。
　　**一、**充分认识新形势下做好人口计生与扶贫开发相结合工作的重要性
　　（一）人口计生与扶贫开发工作取得显著成绩。人口是影响经济社会发展的关键因素，关系着贫困地区脱贫致富和全面协调可持续发展。贫困地区脱贫致富，有利于群众生育观念的转变和计划生育工作的深入开展。改革开放以来，在党中央、国务院的正确领导下，各级人口计生、扶贫开发部门认真贯彻落实科学发展观，将人口计生与扶贫开发工作结合起来，在扶贫开发政策、项目上对计划生育扶贫对象予以优先优待，创造了“少生快富”工程、长效节育措施奖励等行之有效的经验，人口计生和扶贫开发工作取得了显著成绩，贫困地区生育水平逐步下降，人口过快增长的势头得到有效控制，统筹解决人口问题迈出重要步伐，缓解了人口对资源、环境的压力，促进了贫困地区经济发展、社会和谐和民生改善；一大批计划生育扶贫对象脱贫致富，农村贫困人口大幅减少，收入水平稳步提高，农村居民生存和温饱问题基本解决。
　　（二）加强人口计生与扶贫开发相结合工作具有重要意义。当前，我国人口发展正处于重大转折期，人口素质、结构、分布正成为影响发展的主要因素；扶贫开发任务十分艰巨，扶贫对象规模大，相对贫困问题凸显。人口多、增长快、素质低仍是制约贫困地区脱贫致富、群众生活水平提高的重要因素。贫困地区人口计生工作关系着全国稳定低生育水平、统筹解决人口问题的大局。加强人口计生与扶贫开发相结合工作，是实现和稳定贫困地区低生育水平，全面提升人口计生工作水平的重大举措；是促进贫困家庭脱贫致富，实现贫困地区经济社会更好更快发展的重要途径；是促进人口与经济、社会、资源、环境协调和可持续发展的迫切要求；是推动人口计生与扶贫开发事业共同进步的必由之路，必须坚持不懈地抓好落实。

　　**二、**明确人口计生与扶贫开发相结合工作的指导思想、目标任务和基本原则
　　（三）指导思想。
　　以科学发展观为指导，认真贯彻《[中华人民共和国国民经济和社会发展第十二个五年规划纲要](https://www.pkulaw.com/chl/603c7a7f2158b52bbdfb.html)》和《中国农村扶贫开发纲要（2011-2020年）》精神，适应转变经济发展方式、保障和改善民生、增强扶贫对象自我发展能力的新形势，加大对贫困地区人口计生工作的支持力度，加大对计划生育扶贫对象的扶持力度，实现和稳定低生育水平，提高人口素质，优化人口结构，引导人口合理分布，促进贫困地区人口与经济、社会、资源、环境协调发展和可持续发展。
　　（四）目标任务。
　　制定完善人口计生与扶贫开发相结合的政策措施，建立健全党政主导、部门配合、社会协同的工作机制。到2015年，力争使国家扶贫开发工作重点县（以下简称重点县）平均人口自然增长率控制在8‰以内，总和生育率控制在1.8左右。计划生育扶贫对象大幅减少，家庭发展能力明显增强，群众生活水平明显提高。到2020年，重点县低生育水平持续稳定，逐步实现人口长期均衡发展。
　　（五）基本原则。
　　一是坚持统筹规划。将人口计生与扶贫开发相结合工作纳入贫困地区经济社会发展总体规划，融入人口计生、扶贫开发工作之中，同部署、同落实、同检查、同考核。
　　二是坚持因地制宜。根据各地实际，分类指导，将连片特困地区作为工作重点，实施针对性和操作性强、群众得实惠的具体政策和项目。
　　三是坚持资源整合。结合社会主义新农村建设、生态建设、环境保护、产业发展工作，统筹专项扶贫、行业扶贫及社会扶贫，推动社会各界支持贫困地区人口计生工作和关怀帮助计划生育扶贫对象。

　　**三、**完善人口计生与扶贫开发相结合的政策措施
　　（六）健全人口计生利益导向政策体系。进一步完善农村计划生育家庭奖励扶助制度、“少生快富”工程和计划生育家庭特别扶助制度，提高标准，扩大范围。在工作基础比较薄弱、生育水平较高的连片特困地区，推广实施诚信计生奖励政策。切实贯彻国家助学政策，确保符合条件的计划生育家庭子女及时足额获得资助。鼓励机关、企事业单位、社会团体和个人开展对农村计划生育家庭帮困扶持。大力推进“生育关怀行动”、“幸福工程”。开展关怀关爱农村留守儿童、留守老人的活动。
　　（七）提高出生人口素质。普及科学知识，提倡健康科学的生活方式，有针对性地开展宣传教育、健康促进、优生咨询、均衡营养等服务。推进计划生育优质服务，积极创造条件加快在重点县实施免费孕前优生健康检查项目。推动实施贫困地区学龄前儿童营养与健康干预项目。
　　（八）加强基层人口计生服务能力建设。支持重点县人口计生服务机构建设，根据需要适当配备或更新计划生育、优生优育、生殖保健等设备；强化重点县流动服务能力建设。进一步加强重点县人口计生队伍的职业化建设，组织开展专项培训和继续教育，进一步提高干部队伍整体素质；组织发达地区与重点县技术骨干的双向交流培训。
　　（九）深入开展宣传倡导。全面开展婚育新风进万家活动和关爱女孩行动，扎实推进新农村新家庭计划、创建幸福家庭活动，大力传播计划生育、性别平等、优生优育等文明婚育观念，建设新型家庭人口文化。广泛宣传人口计生法律法规和政策，提高群众知晓率，引导更多群众遵纪守法、少生优生。扎实推进人口计生基层群众自治，广泛开展诚信计生工作、阳光计生行动，动员组织群众参与人口计生服务管理。
　　（十）加大对人口计生工作做得比较好的贫困村支持力度。扶贫开发、农田基本建设、水利设施建设、土地开发、新农村建设等项目、资金，要向人口计生工作做得比较好的贫困村倾斜。
　　（十一）加大对计划生育扶贫对象的扶持力度。实施易地扶贫搬迁、以工代赈、产业扶贫、“雨露计划”、就业促进等专项扶贫政策或项目时，要优先优待符合条件的计划生育扶贫对象；发展特色产业、完善基础设施、可再生能源开发利用、危房改造、贴息贷款、定点扶贫、“兴边富民”行动等政策或项目，要向计划生育扶贫对象倾斜，支持计划生育扶贫对象调整产业结构，因地制宜发展种植、养殖、农产品加工、手工艺品制作等产业，提高家庭发展能力。

　　**四、**健全人口计生与扶贫开发相结合工作机制
　　（十二）加强组织领导。各级人口计生、扶贫开发部门要积极做好党委、政府的参谋助手，健全各级人口计生和扶贫开发工作领导机构，完善人口计生目标管理责任制和扶贫开发工作责任制，坚定不移地抓好人口计生与扶贫开发相结合工作。要将人口计生与扶贫开发相结合工作纳入重点县人口计生、扶贫开发部门及相关机构的考核范围，完善考核制度。对在工作中作出突出贡献的组织和个人，按照国家有关规定给予表彰奖励。
　　（十三）强化部门配合。县级以上政府人口计生部门要主动会同扶贫开发部门，研究制定人口计生与扶贫开发相结合的具体政策措施。扶贫开发部门要统筹安排扶贫项目，采取有力措施促进计划生育扶贫对象增加收入；拟定重要政策措施和项目方案，要提前征求人口计生部门的意见。县级以下政府扶贫开发部门或机构在工作中发现政策外怀孕、生育的扶贫对象，要及时与同级人口计生部门或机构沟通，并配合做好宣传动员工作。县级以上政府人口计生、扶贫开发部门要指定专人负责人口计生与扶贫开发相结合工作，会同相关部门定期组织对政策措施执行情况的检查、督导，共同研究解决工作中存在的问题。
　　（十四）共享信息资源。人口计生部门要帮助扶贫开发部门在建档立卡、扶贫对象识别过程中，准确把握贫困家庭人口信息。扶贫开发部门要与人口计生部门及时沟通扶贫对象信息。重点县全员人口数据库要增加家庭贫困状况的有关信息，扶贫对象档案要增加是否为计划生育户、是否落实节育措施等内容。做好全员人口数据库与贫困农户信息管理系统的衔接，实现信息互通共享，及时更新。
　　（十五）推进改革创新。适应加强和创新社会管理的新要求，更加注重利益导向，更加注重服务关怀，更加注重宣传倡导，积极推进相关惠民强农政策与人口计生政策衔接、配合，切实转变贫困地区工作思路和方式。要把典型示范与普遍要求结合起来，积极探索创新人口计生与扶贫开发相结合的新项目和新载体。树立和培养一批实行计划生育的致富典型，营造良好的舆论氛围。加强调查研究，总结推广成功经验。
　　各地要根据本意见，结合实际进一步明确人口计生与扶贫开发相结合的政策措施，制定具体实施方案。

© 北大法宝：（[www.pkulaw.com](https://www.pkulaw.com/)) 专业提供法律信息、法学知识和法律软件领域各类解决方案。北大法宝为您提供丰富的参考资料，正式引用法规条文时请与标准文本核对。
欢迎查看所有[产品和服务](https://www.pkulaw.com/helps/2.html)。法宝快讯：[如何快速找到您需要的检索结果？](https://www.pkulaw.com/helps/70.html)[法宝](https://www.pkulaw.com/helps/69.html)V6有何新特色？

扫描二维码阅读原文

**原文链接：**<https://www.pkulaw.com/chl/2bd75aa1e5766863bdfb.html>
